# Important Factors in Remote Experiential Education

**DOI:** 10.3390/pharmacy10050122

**Published:** 2022-09-24

**Authors:** Jennifer L. Prisco, Jennifer D. Goldman, Tewodros Eguale, Nicole Carace

**Affiliations:** 1 Massachusetts College of Pharmacy and Health Sciences, School of Pharmacy, Boston, MA 02115, USA; 2 Massachusetts College of Pharmacy and Health Sciences, School of Pharmacy, Manchester, NH 03101, USA

**Keywords:** experiential education, remote learning, virtual education, pharmacy

## Abstract

Onsite and in-person experiential education has been well established to prepare practice-ready healthcare professionals, such as pharmacists. From COVID-19, the integration of remote educational delivery has occurred. As healthcare disciplines adjust to new experiential styles and innovate traditional methods, this paper highlights key areas for remote experiential education that can influence student experiences. Factors that are of importance to continuous quality improvement are described. A survey, utilizing the cloud-based software platform Qualtrics^®^ headquartered in the United States, was developed to evaluate whether remote rotation delivery was comparable to traditional onsite experiential education, to assist with quality improvement for virtual experiential education, and to ensure the redesigned educational model meets accreditation standards for two schools of pharmacy. Numerous factors including work, time zone, Office of Experiential Education and preceptor responsiveness, and technology, were examined. Chi-Square test, *t*-test for proportions and odds ratios were utilized to evaluate results. Students with technology concerns throughout a remote rotation had a more than two-fold increase in identifying the virtual experience as worse than most/all other in-person rotations (*p* = 0.01). Preceptor responsiveness to questions and concerns significantly impact student perceptions of educational quality (*p* < 0.05). The majority of students perceived remote experiential education is equal to onsite experiences. Since continuous quality improvement is required by pharmacy accreditors and many other healthcare programs offering clinical opportunities, identifying factors is of importance to make future interventions in the remote experiential education delivery. This type of experiential learning became essential with COVID-19 impacting onsite clinical placements, and information can be used across health science disciplines at large.

## 1. Background

The majority of advanced clinical experiences for healthcare students has been in-person, onsite contact time. Due to the pandemic stemmed from coronavirus (COVID-19), a modification to incorporate high levels of remote/virtual experiential education is warranted. Schools must rise to these unprecedented challenges.

Advanced Pharmacy Practice Experience (APPE) onsite and in-person education has been well established to prepare practice-ready pharmacists within a Doctor of Pharmacy (PharmD) program [1]. During this phase of the program, students build competency in the application of the Pharmacists’ Patient Care Process (PPCP) [1,2]. The Accreditation Council for Pharmacy Education (ACPE) in the United States requires students have 1440 advanced experiential hours providing direct patient care with various patient populations and interprofessional education [3,4,5,6].

All pharmacy students at Massachusetts College of Pharmacy and Health Sciences across three campuses are required to complete six advanced rotations in the last year of their program, including inpatient general medicine, ambulatory care, community pharmacy, and hospital/health system pharmacy rotations. Each rotation is 6 weeks in length and 40 hours per week. A combination of full-time pharmacist faculty and adjunct faculty are used to conduct experiential rotations. In March 2020, the majority of hospital, health-system, and ambulatory experiential sites throughout New England were no longer able to accommodate students in person, due to becoming a hot spot at this time for COVID-19 and drastic increases in hospitalizations. As 489 students in the PharmD Class of 2020 across three campuses at two separately accredited pharmacy programs needed at least one last advanced rotation to graduate, massive efforts by the centralized Office of Experiential Education (OEE), in partnership with full-time faculty, worked to devise and accommodate remote experiential education. With the remote delivery, only full-time faculty or adjunct faculty with high awareness of ACPE accreditation standards were utilized as a preceptor in APPE core rotations. Students were required to complete the same core rotation type that they were initially assigned to for this last APPE block, unless a prior advanced elective rotation could account for the remaining APPE core rotation. For example, if a student completed an inpatient specialty elective that could account for a core inpatient general medicine rotation, the prior elective was adjusted to document the core rotation completion, and the last APPE block became a remote elective. Students had their preceptor changed to full-time faculty or adjunct faculty with high levels of ACPE awareness for this last core APPE block. Electives were reassigned when needed if a preceptor could not pivot to a remote format. Adjusted electives were based on what could be offered and adapted remotely such as digital health, informatics and drug information opportunities.

By previously completing five 6-week APPE rotations each onsite throughout the 2019–2020 academic year, these students had significant onsite experiential opportunities in which to compare to a remote experience. A survey, utilizing the cloud-based software platform Qualtrics^®^ headquartered in the United States, was developed to evaluate whether the new rotation delivery was comparable to traditional onsite experiential education, to assist with quality improvement for virtual delivery in the next academic year, and to ensure experiential education meets accreditation standards. Accreditation requires pharmacy programs to administer Curriculum Quality Surveys to evaluate whether experiential rotations are of high quality; whether preceptors provided individualized instruction, guidance and evaluation; and whether information technology resources and educational resources provided were conducive to learning [7].

OEE hypothesized that the majority of students would be of the opinion that the remote experience would be equal to most/all other rotations they previously had in-person. As healthcare disciplines nationwide adjust to new experiential delivery styles [8,9,10,11,12] and integrate flexibility allotted by accreditors, [1] the aim of this paper is to describe the survey results and to highlight key areas for remote delivery of experiential education that can influence student experiences across all health care programs.

## 2. Approach

The majority of the 39-question survey was linked to accreditation standards and continuous quality improvement (CQI). Upon completion of the remote rotation, students answered demographic and basic information questions including rotation type, living arrangements, and paid work experience while attending the remote rotation. Questions from a section related to the virtual rotation experience to guide CQI, student support, and student satisfaction, all of which are areas highlighted within ACPE accreditation standards or guidance documents, were analyzed [3,13]. Eleven questions were asked in which students rated their level of agreement or disagreement on a five-point Likert scale. Three of the eleven questions were linked to accreditation standards, six questions were linked to factors that could impact the virtual delivery format and were considered CQI-related questions, and two questions asked students to evaluate responsiveness of their preceptor and OEE. The number of preceptors the students interacted with was also evaluated, as rotations in the remote format were adjusted to have team-based precepting for about half the core rotations. In this adjusted model, there was still the expectation that students should receive individualized instruction.

A student perception question was asked regarding the virtual rotation quality compared to previous onsite rotations. This question was cross tabulated with other survey questions using Chi-Square test, *t*-test for proportions and odds ratios. Students could provide free text information. This methodology allowed for analysis of factors that impact student perceptions and required accreditation components.

## 3. Evaluation

Table 1 shows the number of students, rotation type, and survey response rates for the remote APPE across the three campuses. The majority of students attending this remote rotation (76.7%, *n* = 375) responded to the survey, and 355 students completed the survey in its entirety. The percent of students who responded to the survey (*n* = 375), when compared to the percent of students in each rotation type during the remote rotation (*n* = 489), is within 0.2%–1.8% of each other for all rotation types.

Of the 355 fully responding students, 48.5% completed paid work experience while also completing the remote rotation. The majority worked similar hours to pre-pandemic. Approximately half (49.3%) of the remote rotations were conducted in a group format with 2+ preceptors collaborating together.

Highlights from the eleven Likert-scale questions in Table 2 include 39.1% of respondents (*n* = 139) somewhat to strongly agreed they had technology concerns throughout the rotation and 15% (*n* = 53) somewhat to strongly agreed that time zone differences were a concern for this rotation. The majority of respondents somewhat to strongly agreed that their experiential and academic expectations were met for this rotation (91.5%) and they received individualized attention from their preceptor during the rotation that met their expectations (92.4%). For this block, the majority of respondents somewhat to strongly agreed their preceptor (94%, *n* = 303), and OEE (66%, *n* = 156) was responsive to their questions and concerns.

Students evaluated the remote rotation quality compared to their on-site rotations. This question resulted in 16.6% (*n* = 59) of students being of the opinion that the virtual rotation was better than most/all other rotations, 67.6% (*n* = 240) reported the virtual rotation as equal to most/all other rotations, and 15.8% (*n* = 56) viewed this rotation as worse than most/all other rotations.

Summarizing Table 3, students working and the amount of hours worked, time zone differences, various group formats that preceptors utilized, and responsiveness from OEE, did not have a statistically significant impact on the student’s perception for this rotation being better than, equal to, or worse than most/all other prior rotations (*p* > 0.05 for all). The preceptor’s response to questions and concerns was a significant finding that impacted student perceptions of the quality when using Chi-Square testing (*p* < 0.05). Of the students who did not strongly agree that their preceptor was responsive to questions and concerns, 36.7% had the opinion that the remote rotation was of worse than most/all other rotations (*n* = 49). In contrast, if a student strongly agreed their preceptor was responsive, 12.4% had the opinion that the remote rotation was of worse than most/all other rotations (*n* = 275).

Students who had technology concerns throughout this rotation had a more than two-fold increase in identifying the virtual experience as worse than most/all other rotations (odds ratio 2.24, 95% CI 1.20, 4.16, *p* = 0.01) compared to students with no technology concerns; 23% of students with technology concerns evaluated the virtual session as worse than most/all other rotations compared to 11.8% of students with no technology concern.

Based on these results, the majority of students perceived remote experiential education is equal to or better than their onsite experiences.

## 4. Implications

In March 2020, there were no vaccines for COVID-19, there were shortages on personal protective equipment (PPE), and there were many unknowns. There are now vaccinations and PPE readily available, and yet there is still a need to be prepared to offer remote clinical education with ongoing COVID-19 surges, future natural disasters, or other.

Key findings that impact remote experiential education include the following: (1) technology issues play a principle role in a student’s satisfaction level, and needs to be addressed effectively, and (2) preceptor responsiveness to students’ questions and concerns impact student perceptions. Interventions made with this information include focusing on technology referral support during remote experiential delivery and providing preceptor development with an emphasis on responding to student questions and concerns. For technology, students had to adapt with the means they had during this APPE block, and no school-issued laptop or communication device was distributed at large. Within the 6 weeks of this rotation, the school pivoted to ensure laptop or communication devices could be available for students in need, and also looked into internet accessibility options such as a hotspot device to provide to students who were without one. These discoveries are applicable to any clinical placement that integrates remote experiential education.

Historically with onsite rotations, the program used a model where one specific preceptor is the point person for each student. In the remote setting as OEE and faculty worked together to shift rotations, many faculty elected to collaborate together in teams of 2, 3, or 4+ preceptors for their assigned students. This teamwork expanded student opportunities to collaborate beyond one person, expanded direct patient care opportunities, and allowed preceptors to diversify topics with the unanticipated remote offering. Knowing team-based precepting had benefits and did not negatively impact student experiences, it is a model to consider for future use.

For students that somewhat to strongly disagreed OEE was responsive to questions and concerns, students did not provide any comments on how to improve the experience. Anecdotally through emails received from students, there was dissatisfaction from some students on not receiving a reassignment to a top rotation pick, on being assigned to a preceptor not of their choosing, or on being in a remote experiential placement altogether. Given the environment and loss of onsite clinical placements, some of the dissatisfaction could not be avoided. Communication with students during these times is crucial.

By evaluating factors potentially impacting rotations (work, time zone differences, preceptor responsiveness, etc.) and student perceptions of the remote rotation being better than, equal to, or worse than onsite experiential education, this allowed the program to pinpoint factors that can significantly influence student perceptions of the remote quality. Since CQI and evaluating student satisfaction is required by accreditors such as ACPE, identifying factors is important to make future interventions in the remote experiential delivery. This project included a large sample size for identification and evaluation, allowing for development and improvement of future remote rotations if needed. Limitations include awareness that by not completing a virtual rotation, students would be ineligible to graduate on time due to experiential rotation displacements brought forth by the pandemic. To address this limitation, the cross-tabulation method was utilized to evaluate particular experiential factors that most significantly impacted student perceptions. Further, although the authors can confirm the majority of students got the same APPE type in the remote format and the majority of students got a preceptor they had ranked as a top 10 preceptor, the exact breakdown for the number of changes made is not readily available. A limitation of this is not having the information to cross-tabulate as a factor for satisfaction level. While the study was limited to pharmacy students only, what was learned can be translated to all types of academic clinical rotations.

## Figures and Tables

**Table 1:** Students completing each rotation type remotely.

	Students in Each Rotation		Students Who Responded to the Survey		Response Rate
Virtual rotation type completed	Count (*n*)	Percent in block 8	Count (*n*)	Percent in block 8	Percent in block 8
APPE Ambulatory Patient Care	82	16.8%	64	17.1%	78%
APPE Community Pharmacy	63	12.9%	50	13.3%	79.4%
APPE Elective	191	39.1%	140	37.3%	73.3%
APPE Institutional (Hospital/Health System Pharmacy)	71	14.5%	55	14.7%	77.5%
APPE Internal Medicine (Inpatient General Medicine)	82	16.8%	66	17.6%	80.5%
Total	489	100.0%	375	100%	76.7%

**Table 2:** Survey questions related to accreditation standards, continuous quality improvement, and responsiveness from preceptors and the Office of Experiential Education.

	Strongly Agree %	Somewhat Agree %	Neither Agree Nor Disagree %	Somewhat Disagree %	Strongly Disagree %
My experiential and academic expectations were met for this specific rotation type ^A^	56.9%	34.6%	4.5%	3.1%	0.8%
During this rotation, I completed direct patient care activities (important: please use direct patient care activities as defined by the ACPE best practice list that you had to document at the beginning of this rotation) ^B^	28.7%	23.1%	26.8%	9.6%	11.8%
During this rotation, I completed at least one activity, assignment, or topic discussion related to COVID-19/coronavirus ^A^	63.7%	16.3%	6.2%	5.6%	8.2%
I received individualized attention from my preceptor during this rotation that met my expectations ^B^	73.5%	18.9%	3.9%	2.3%	1.4%
For the delivery of this rotation, I enjoyed the virtual format ^A^	46.2%	29.9%	13.0%	6.8%	4.2%
I prefer to have a letter/numeric grade for this virtual rotation rather than a pass/fail grade ^A^	63.1%	14.1%	15.2%	5.1%	2.5%
I had technology concerns throughout this rotation ^B^	11.8%	27.3%	15.5%	15.2%	30.1%
Time zone differences were a concern for this rotation ^A^	6.5%	8.5%	16.1%	9.3%	59.7%
The Office of Experiential Education was helpful in the rotation reassignment process for block 8 ^A^	45.4%	20.8%	27.6%	3.4%	2.8%
The Office of Experiential Education was responsive to my questions and concerns for this rotation block ^C^	46.0%	19.8%	24.9%	5.1%	4.2%
My preceptor was responsive to my questions and concerns for this rotation block ^C^	84.9%	8.6%	4.3%	0.3%	1.9%

A Questions aligned to continuous quality improvement for the remote rotation. B Questions aligned to ACPE accreditation standards for the remote rotation. C Questions aligned to responsiveness for questions/concerns in the remote rotation.

**Table 3:** Chi-squared test: cross tabulation questions to whether the virtual rotation was better than, equal to, or worse than most/all other APPE rotations that were onsite.

		Cross Tabulation Responses (*n*)			
Question	Response Choices (*n*, %)	Remote APPE Was Better than Most/All Other Rotations	Remote APPE Was Equal to Most/All Other Rotations	Remote APPE Was Worse than Most/All Other Rotations	*p*-Value
Did you also complete paid work experience while completing APPE virtually?	Yes (172, 48.5%)	29	117	26	*p* > 0.05
	No (183, 51.5%)	30	123	30	
Compared to the rest of the APPE year, the number of hours per week that I worked at my job during this remote APPE	Increased (54, 31.4%)	9	36	9	*p* > 0.05
	Stayed the same (95, 55.2%)	17	64	14	
	Decreased (23, 13.4%)	3	17	3	
Time zone differences were a concern for this rotation	Strongly agree (23, 6.5%)	4	14	5	*p* > 0.05
	Somewhat agree (30, 8.5%)	5	19	6	
	Neither agree nor disagree (57, 16.1%)	8	40	9	
	Somewhat disagree (33, 9.3%)	6	24	3	
	Strongly disagree (212, 59.7%)	36	143	33	
I had technology concerns throughout this rotation	Strongly agree (42, 11.8%)	4	26	12	*p* > 0.05
	Somewhat agree (97, 27.3%)	12	65	20	
	Neither agree nor disagree (55, 15.5%)	12	38	5	
	Somewhat disagree (54, 15.2%)	9	40	5	
	Strongly disagree (107, 30.1%)	22	71	14	
My remote rotation was primarily conducted in the following format	I was primarily with one preceptor for the entire rotation (180, 50.7%)	30	120	30	*p* > 0.05
	I collaborated with 2–3 preceptors and their students throughout the entire rotation on various activities (67, 18.9%)	15	43	9	
	I collaborated with 4 or more preceptors and their students throughout the entire rotation on various activities (108, 30.4%)	14	77	17	
The Office of Experiential Education was responsive to my questions and concerns for this rotation block	Strongly agree (109, 46.0%)	20	77	12	*p* > 0.05
	Somewhat agree (47, 19.8%)	6	35	6	
	Neither agree nor disagree (59, 24.9%)	9	41	9	
	Somewhat disagree (12, 5.1%)	2	6	4	
	Strongly disagree (10, 4.2%)	0	9	1	
	Not applicable (*n* = 118)				
My preceptor was responsive to my questions and concerns for this rotation block	Strongly agree (275, 84.9%)	50	191	34	*p* < 0.05
	Somewhat agree (28, 8.6%)	1	17	10	
	Neither agree nor disagree (14, 4.3%)	1	7	6	
	Somewhat disagree (1, 0.3%)	0	1	0	
	Strongly disagree (6, 1.9%)	1	3	2	
	Not applicable (*n* = 31)				

## Data Availability

The data presented in this study are available on request from the corresponding author. The data are not publicly available due to academic privacy.
